# The role of serum procalcitonin and CRP levels in determining of etiology and outcome in acute exacerbations of COPD

**DOI:** 10.1186/2197-425X-3-S1-A796

**Published:** 2015-10-01

**Authors:** O Ceylan, I Ucgun, T Us, N Kasifoglu, A Kiremitci, F Demircan, S Erginel

**Affiliations:** Eskisehir Osmangazi University Medical Faculty, Department of Chest Dis., Eskisehir, Turkey; Eskisehir Osmangazi University Medical Faculty, Department of Microbiology, Eskisehir, Turkey

## Introduction

This study was conducted to investigate the role of serum procalcitonin and CRP levels in determining etiology, treatment and prognosis in COPD patients with acute exacerbation.

## Methods

Fifty-eight hospitalized COPD patients with acute exacerbation were included in the study between January 2008 and March 2011. The diagnosis of COPD was made according to the GOLD guidelines. Serum CRP and procalcitonin (PCT) levels in the first day of hospitalization and then one month later (in a stable period) were studied from all COPD patients. Patients were classified into 4 groups according to the severity of disease and divided into 2 groups according to the PCT levels

( < 0.2 ng/dl and > 0.2 ng/dl).

## Results

Four (7%) patients were female and 54 (93%) were male. The mean age was 68.1 ± 10.1. Pathogenic bacterial growth was detected in 16 sputum or bronchial lavage samples. MRSA was found in 3 samples, P. aeruginosa in 3, S. maltophilia in 3, and Enterobacter species was detected in 2 samples. Chlamydia pneumoniae serology was positive in 16 (29%) patients. M. pneumoniae was positive in 8 (15%) patients. The rate of M. pneumoniae was highest (40%) in the severe exacerbation group. PCT level was significantly higher in the Chlamydia positive group (40% vs. 22%). The mean CRP level of COPD patients was 6.7 mg/l in acute attack, and 2.0 mg/l in the stable period. The mean PCT level was 0.50 ng/l in acute attack, and 0.09 ng/l in the stable period. Serum PCT level was found as 0.44 ng/l in severe COPD group, and 0.2 ng/ml in the others (p = 0.02). 27 patients with acute exacerbation of COPD were diagnosed with sepsis. Six patients in the study group died. Mortality rate was higher in high PCT group (13.3% vs. 2.5%).

## Conclusions

PCT level can help for determining acute attack and etiology in COPD patients.

